# The gibberellic acid derived from the plastidial MEP pathway is involved in the accumulation of *Bamboo mosaic virus*

**DOI:** 10.1111/nph.18210

**Published:** 2022-06-22

**Authors:** Ying‐Ping Huang, I‐Hsuan Chen, Yu‐Shun Kao, Yau‐Heiu Hsu, Ching‐Hsiu Tsai

**Affiliations:** ^1^ Graduate Institute of Biotechnology National Chung Hsing University Taichung 402 Taiwan; ^2^ Advaced Plant Biotechnology Center National Chung Hsing University Taichung 402 Taiwan

**Keywords:** 2‐*C*‐methyl‐d‐erythritol‐4‐phosphate (MEP) pathway, bamboo mosaic virus (BaMV), geranylgeranyl pyrophosphate (GGPP) synthases, gibberellic acid (GA_3_) synthesis pathway, isopentenyl pyrophosphate, *Nicotiana benthamiana*

## Abstract

- A gene upregulated in *Nicotiana benthamiana* after *Bamboo mosaic virus* (BaMV) infection was revealed as 1‐deoxy‐d‐xylulose‐5‐phosphate reductoisomerase (NbDXR). DXR is the key enzyme in the 2‐*C*‐methyl‐d‐erythritol‐4‐phosphate (MEP) pathway that catalyzes the conversion of 1‐deoxy‐d‐xylulose 5‐phosphate to 2‐*C*‐methyl‐d‐erythritol‐4‐phosphate.
- Knockdown and overexpression of *NbDXR* followed by BaMV inoculation revealed that NbDXR is involved in BaMV accumulation. Treating leaves with fosmidomycin, an inhibitor of DXR function, reduced BaMV accumulation. Subcellular localization confirmed that DXR is a chloroplast‐localized protein by confocal microscopy. Furthermore, knockdown of 1‐hydroxy‐2‐methyl‐2‐(*E*)‐butenyl‐4‐diphosphate reductase, one of the enzymes in the MEP pathway, also reduced BaMV accumulation. The accumulation of BaMV increased significantly in protoplasts treated with isopentenyl pyrophosphate. Thus, the metabolites of the MEP pathway could be involved in BaMV infection.
- To identify the critical components involved in BaMV accumulation, we knocked down the crucial enzyme of isoprenoid synthesis, NbGGPPS11 or NbGGPPS2. Only NbGGPPS2 was involved in BaMV infection. The geranylgeranyl pyrophosphate (GGPP) synthesized by NbGGPPS2 is known for gibberellin synthesis. We confirmed this result by supplying gibberellic acid exogenously on leaves, which increased BaMV accumulation.
- The *de novo* synthesis of gibberellic acid could assist BaMV accumulation.

## Introduction

A virus cannot complete its infection cycle without support from its associated host. Thus, host factors are crucial for participating in most steps of virus infection (Whitham & Wang, 2004; Medina‐Puche & Lozano‐Duran, 2019), including viral protein translation (Thivierge *et al*., 2005; Nicholson & White, 2011), virus replication (Ahlquist *et al*., 2003; Noueiry *et al*., 2003; Laliberte & Sanfacon, 2010; Ishibashi & Ishikawa, 2016), intracellular tracking (Niehl & Heinlein, 2011; Schoelz *et al*., 2011; Cheng, 2017), and cell‐to‐cell movement in plants (Lucas, 2006; Benitez‐Alfonso *et al*., 2010; Liou *et al*., 2015). Moreover, host proteins can also impair virus infection (Lin *et al*., 2012; Huang *et al*., 2017b; Chen *et al*., 2018, 2019; Garcia‐Ruiz, 2019). Hence, identifying the roles of these host proteins involved in virus infection could uncover the viral infection mechanism. A few approaches have been used to screen for host factor candidates, including transposon tagging, screening the mutants in yeast or Arabidopsis, and complementary DNA (cDNA)‐amplified fragment length polymorphism (AFLP) combined with virus‐induced gene silencing (VIGS) (Dinesh‐Kumar *et al*., 1995; Panavas *et al*., 2005; Cheng *et al*., 2010).

*Bamboo mosaic virus* (BaMV) is a positive‐sense RNA virus containing one single‐stranded genome belonging to the *Potexvirus* of Alphaflexiviridae. The 5′ and the 3′‐ends of the genome are m^7^GpppG‐capped and poly(A)‐tailed, respectively (Lin *et al*., 1994; Chen *et al*., 2017b). The RNA genome contains five open reading frames (ORFs). ORF1 encodes a 155 kDa replicase of three domains: a capping enzyme domain harboring methyltransferase and *S*‐adenosylmethionine‐dependent guanylyltransferase activity (Li *et al*., 2001a; Huang *et al*., 2004, 2005), a helicase‐like domain with RNA 5′‐triphosphatase activity (Li *et al*., 2001b), and a polymerase domain with RNA‐dependent RNA polymerase activity (Li *et al*., 1998). ORF2 to 4, known as the triple gene block (TGB), encode the viral movement proteins TGBp1 to TGBp3 (Lin *et al*., 2004, 2006). ORF5 encodes the 25 kDa coat protein (CP) functioning in virus encapsidation, movement, and symptom development (Lin *et al*., 1994; Lan *et al*., 2010; Lee *et al*., 2011; Hung *et al*., 2014).

cDNA‐AFLP was used to screen differentially expressed genes in *Nicotiana benthamiana* after BaMV infection (Cheng *et al*., 2010). Host proteins were identified to be important for BaMV genomic RNA synthesis (e.g. a glutathione transferase NbGSTU4 (Chen *et al*., 2013), a small Rab GTPase NbRabG3f (Huang *et al*., 2016), a carbonic anhydrase NbCA (Chen *et al*., 2017c), the autophagy‐related proteins NbATGs (Huang *et al*., 2019), and the nonspecific lipid transfer protein 1 NbLTP1 (Chiu *et al*., 2020)) and movement (e.g. a small Rab GTPase‐activation protein NbRabGAP1 (Huang *et al*., 2013), a Ser/Thr kinase‐like protein NbSTKL (Cheng *et al*., 2013), an elicitor‐inducible leucine‐rich repeat receptor‐like protein NbEILP (Chen *et al*., 2017a), and a small Rab GTPase NbRabF1 (Huang *et al*., 2020)). By contrast, differentially expressed genes could also have negative roles in BaMV infection (e.g. a plasma membrane‐associated cation‐binding protein 1‐like protein NbPCaP1L (Chen *et al*., 2017a), a thioredoxin NbTRXh2 (Chen *et al*., 2018), and an E3 ubiquitin ligase NbUbE3R1 (Chen *et al*., 2019)).

One of the differentially expressed genes, 1‐deoxy‐d‐xylulose‐5‐phosphate reductoisomerase (DXR) (Cheng *et al*., 2010) encodes the key enzyme DXR in the 2‐*C*‐methyl‐d‐erythritol 4‐phosphate (MEP) pathway that converts 1‐deoxy‐d‐xylulose‐5‐phosphate (DXP) to MEP and regulates the concentration of isoprenoid. Overexpression of *Nicotiana tabacum* DXR (NtDXR) in tobacco plants enhanced the accumulation of downstream products, including Chl, carotenoid, and isoprenoids (Hasunuma *et al*., 2008). Disruption of the *DXR* gene in *Arabidopsis* could result in albino and dwarf traits (Xing *et al*., 2010). DXR, *c*. 51 kDa in tobacco plants, is a nuclear‐encoded plastidial enzyme containing an N‐terminal chloroplast transit peptide carrying a putative 14‐3‐3 binding motif (Fung *et al*., 2010). AtDXR was localized in chloroplast stroma by electron microscopy (Carretero‐Paulet *et al*., 2002).

The product of MEP converted by DXR is gone through a series of enzymes (4‐diphosphocytidyl‐2‐*C*‐methyl‐d‐erythritol synthase, 4‐diphosphocytidyl‐2‐*C*‐methylerythritol kinase (CMK), 2‐*C*‐methyl‐d‐erythritol 2,4‐cyclodiphosphate synthase, 4‐hydroxy‐3‐methylbut‐2‐en‐1‐yl diphosphate synthase, and 1‐hydroxy‐2‐methyl‐butenyl 4‐diphosphate reductase (HDR)) to produce the end‐product of the MEP pathway, isopentenyl diphosphate (IPP) (Banerjee & Sharkey, 2014). IPP is a C_5_‐unit, a central compound for synthesizing sterols, dolichols (cell wall biosynthesis), terpenes, quinine (electron carrier), carotenoids, and Chls (Lichtenthaler, 1999). The synthesis of IPP can involve the cytoplasmic acetate/mevalonate (MVA) and plastidial MEP, or non‐MVA, pathways (Nagegowda, 2010). The volatile terpenoids synthesized via these pathways could play a defensive role against pathogens and herbivores (Arimura *et al*., 2009; Nagegowda, 2010).

A few phytohormones are synthesized from the follow‐up reactions using IPP as the precursor. These phytohomones regulate most plant physiological processes and are also involved in plant defense against viruses (Santner & Estelle, 2009; Alazem & Lin, 2015; Collum & Culver, 2016; Zhao & Li, 2021). Gibberellic acid (GA) is a plant hormone that is essential for many developmental processes *in planta*, including seed germination, organ elongation and expansion, trichome development, transition from vegetative to reproductive growth, and flower/seed/fruit development (Achard & Genschik, 2009; Daviere & Achard, 2013; Hedden & Sponsel, 2015). The precursor of GA biosynthesis is converting the end‐product of the MEP pathway, IPP, to geranylgeranyl pyrophosphate (GGPP) by GGPP synthase (GGPPS) (Hedden & Sponsel, 2015). The follow‐up reactions involve converting GGPP to *ent*‐kaurene by two steps of cyclization with *ent*‐copalys diphosphate synthase and *ent*‐kaurene synthase (KS) (Binenbaum *et al*., 2018). All these reactions for the synthesis of GA precursors occur in the chloroplast. The formation of bioactive GAs occurs at the endoplasmic reticulum and cytosol. Although P2 and P7‐2 protein of *Rice dwarf virus* and *Rice black streaked dwarf virus*, respectively, could interact with the GA biosynthesis enzyme or the regulator of GA signaling, the effects on the plant after virus infection were restricted to symptom development (Zhu *et al*., 2005; Tao *et al*., 2017). The virus accumulation level was not affected after applying exogenous GA to infected plants (Zhu *et al*., 2005). Therefore, whether GA and GA signaling have an antiviral function needs to be investigated.

In this study, we revealed how a nuclear‐encoded chloroplast enzyme, *N. benthamiana* DXR (NbDXR), is involved in the accumulation of BaMV. When we traced the critical components of the MEP pathway, we found that the end‐product of the MEP pathway was used to synthesize GA and the accumulation of BaMV was elevated.

## Materials and Methods

### Plants and viruses

The *N. benthamiana* plant was grown using the culture soil (Jiffy substrates) mixed with vermiculite in a 2.5 : 1 ratio in a growth room setting at the 45 μmol m^−2^ s^−1^ light intensity with 16 h : 8 h, light : dark at 28°C. BaMV strain S (Lin & Hsu, 1994) was used for the infection.

### Virus‐induced gene silencing and BaMV inoculation

To knock down the expression of *NbDXR*, *NbCMK*, *NbHDR*, *NbGGPPS11*, *NbGGPPS2*, and *NbKS* in *N. benthamiana*, the tobacco rattle virus (TRV)‐based *Agrobacterium*‐mediated VIGS system was used (Liu *et al*., 2002). The fragment of each target gene was PCR‐amplified with the primer sets listed in Supporting Information Table S1 and cloned into the pGEM‐T Easy vector (Promega), then subcloned into the pTRV2 vector using the *Eco*RI site. *Agrobacterium* containing pTRV1 or pTRV2 derivatives (pTRV2/*luciferase* (luc), /*phytoene desaturase* (PDS), /*NbDXR*, /*NbCMK*, /*NbHDR*, /*NbGGPPS11*, /*NbGGPPS2*, and /*NbKS*) was cultured in 2× yeast extract–tryptone (2YT) medium at 30°C to OD_600_ = 1. The cells were centrifuged at 1478 ***g*** at room temperature (Avanti J‐26 XP; Beckman Coulter Inc., Brea, CA, USA) for 20 min and resuspended in induction buffer (10 mM magnesium chloride (MgCl_2_), 10 mM MES pH5.6, 200 μM acetosyringone) at 30°C for 2 h. The two agrobacteria (containing pTRV1 and pTRV2 derivatives) were mixed in a 1 : 1 ratio and infiltrated onto the second, third, and fourth leaves of 1‐month‐old *N. benthamiana* plants. After 10–14 d, the fourth leaf above the infiltrated leaves was mechanically inoculated with 500 ng of BaMV virion in 10 μl water with carborundum. All the knockdown experiments were done by at least three independent experiments with three plants in each experiment.

### Protoplast isolation and viral RNA inoculation

Approximately 2 g of *N. benthamiana* leaf was digested with 25 ml enzyme solution (0.55 M mannitol‐MES pH 5.7, 0.1% bovine serum albumin, 0.6 mg ml^−1^ pectinase, 12 mg ml^−1^ cellulase) at 25°C for 10–12 h. The cell‐wall‐removed protoplasts were filtered through Miracloth and centrifuged at 26.6 ***g*** for 7 min (Kubota KS‐5000, Osaka, Japan). The spun‐down cells were resuspended in 2 ml of 0.55 M mannitol‐MES (pH 5.7) and layered on top of 2 ml 0.55 M sucrose (pH 5.7). After centrifugation at 26.6 ***g*** for 5 min, the green zone of the interface in each tube containing the protoplasts was transferred to a 5 ml 0.55 M mannitol‐MES tube and spun down at 26.6 ***g*** for 5 min. The spun‐down protoplasts were washed twice with 2 ml of 0.55 M mannitol‐MES. Finally, the protoplasts were suspended in 1 ml 0.55 M mannitol‐MES, and the cell number and quality were inspected under a fluorescence microscope after staining with fluorescein diacetate.

Approximately 5 × 10^5^ protoplasts were inoculated with 500 ng viral RNA by using 40% polyethyleneglycol‐6000. The inoculated protoplasts were centrifuged at 26.6 ***g*** for 4 min and washed with 2 ml 0.55 M mannitol‐MES. Finally, the inoculated protoplasts were incubated at 25°C for 24 or 48 h under constant light. For the GA‐treatment experiment, the culture medium for the BaMV‐inoculated protoplasts was added to with 150 μM GA.

### Total RNA extraction

Total RNA was extracted from 0.2 g mock and 500 ng BaMV virion‐inoculated *N. benthamiana* leaf tissues by using the sodium dodecyl sulfate (SDS)–phenol method (Rio *et al*., 2010). In brief, the inoculated leaf was collected and frozen immediately with liquid nitrogen (N_2_) and pulverized into powder and extracted with the RNA extraction buffer (100 mM Tris hydrochloride pH 8.0, 100 mM lithium chloride, 10 mM EDTA, 1% SDS, and 50% phenol) and incubated at 65°C for 30 s. The RNA‐containing aqueous layer was re‐extracted twice with an equal volume of phenol–chloroform and precipitated with ethanol with sodium acetate. The RNA pellet was washed with 70% ethanol after centrifugation and dissolved in deionized water.

### Reverse transcription and real‐time PCR

To synthesize the first‐strand cDNA, the reaction mixture containing 0.5*–*2 μg total RNA and 4 pmol oligos (dT_25_) was incubated at 70°C for 5 min and kept on ice. The reverse transcription cocktail was added into the reaction containing 5× buffer (Promega), 3 mM MgCl_2_, 0.5 mM deoxynucleoside triphosphate, RNase inhibitor (RNaseOUT; Invitrogen), and reverse transcriptase (Promega). The reaction was incubated at 25°C for 5 min, transferred to 42°C for 1 h, and switched to 72°C for 15 min.

To determine the knockdown efficiency in *NbDXR*‐knockdown plants, the expression of *NbDXR* was determined by using real‐time PCR with the primers shown in Table S1. The 20 μl reaction containing cDNA template (1 μl directly from the previous reaction), 200 nM forward and reverse primers, and 2× KAPA SYBR FAST qPCR Kit Master Mix (KAPA Biosystems Inc., Wilmington, MA, USA) was performed in a TOptical Gradient 96 Real‐Time PCR Thermal Cycler (Biometra, Analytik Jena, Jena, Germany).

### Overexpression of NbDXR‐T7 fusion protein

The full‐length cDNA of *NbDXR* was amplified by PCR with the forward primer NbDXR‐T7F and reverse primer NbDXR‐T7R containing the sequence of T7‐tag (Table S1). The amplified fragment was cloned into the pGEM‐T Easy vector (Promega) and verified by sequencing. The resulting construct pBI‐NbDXR‐T7 was then transformed into the *Agrobacterium tumefaciens* C58C1 strain by electroporation.

*Agrobacterium* containing the pBI‐NbDXR‐T7 or pBI‐HcPro construct was cultured in 2YT medium at 30°C to OD_600_ = 1, then infiltrated into leaves of 2‐month‐old (approximately seven true leaves stage) *N. benthamiana* plants. After 12 h, *c*. 200 ng BaMV virion was mechanically inoculated onto the infiltrated leaves and harvested at 3 d post‐inoculation (dpi).

### Western blot analysis

Total protein was extracted from leaves (0.1 g) ground with liquid N_2_ first or protoplasts (2.5 × 10^5^ cells) with 300 or 40 μl protein extraction buffer (50 mM Tris hydrochloride pH 8.0, 10 mM potassium chloride, 10 mM MgCl_2_, 1 mM EDTA, 20% glycerol, 2% SDS, 10% β‐mercaptoethanol), respectively, vortexed, and boiled for 5 min. After centrifugation, 5 μl of the extract (the supernatant) was loaded on 12% SDS‐polyacrylamide gel with 1× Laemmli buffer.

The gel was split into two parts. The upper part of the gel containing the proteins ≥ 40 kDa was either transferred to a nitrocellulose membrane (Protran BA 85; Thermo Fisher Scientific, Waltham, MA, USA) for actin detection with antibody or stained with Coomassie blue (0.1% w/v Coomassie brilliant blue R‐250, 50% methanol, 10% acetic acid) for 1 h, then destained with 30% methanol and 10% acetic acid for 1 h for Rubisco large subunit (rbcL) detection. The lower part of the gel containing the proteins < 40 kDa was transferred to the membrane. The membrane was incubated with the primary antibody against BaMV coat protein (laboratory generated with 1 : 5000 dilution) or β‐actin (Yao‐Hong Biotechnology Inc., New Taipei City, Taiwan), with 1 : 2000 dilution, and probed with the secondary antibody (affinity purified anti‐rabbit immunoglobulin G with 1 : 10 000 dilution) conjugated with IRDye 800 (Rockland Immunochemicals, Gilbertsville, PA, USA). Finally, the gel and membrane were scanned and quantified by using an Amersham Typhoon Biomolecular Imager (GE Healthcare, Chicago, IL, USA). The banding density of the rbcL on gel or the actin on the membrane was used for normalization.

### Northern blot analysis

Total RNA was extracted from inoculated protoplasts or plants. Approximately 1 μg RNA used in a reaction containing 10 mM phosphate buffer (pH 7.0), 50% dimethyl sulfoxide, and 1 M glyoxal was incubated at 50°C for 1 h. The RNA was separated on 1% agarose gel and transferred to a nylon membrane (Hybond‐N+; GE Healthcare) as described (Weiland & Dreher, 1989; Chen *et al*., 2022). The membrane was probed with 10^7^ cpm [α‐^32^P] UTP‐labeled anti‐BaMV RNA. After hybridization, the membrane was washed; then, the radioactive banding signals on the membrane were detected and quantified using a phosphorimager (Fujifilm BAS 1500, Tokyo, Japan).

### The localization of NbDXR‐orange fluorescent protein

To examine the cellular localization of NbDXR, the full‐length cDNA amplified by PCR with the forward primer NbDXR‐OFPF and reverse primer NbDXR‐OFPR (Table S1) was cloned into the pGEM‐T Easy vector (Promega) and subcloned into the pEpyon vector containing orange fluorescent protein (OFP). The infiltrated leaf was collected at 3 d post‐infiltration, and the protoplasts were isolated from the infiltrated leaf. The cellular localization of NbDXR‐OFP was observed by confocal laser scanning microscopy (FV1000; Olympus, Tokyo, Japan) with an HeNe green laser (543 nm) for OFP and HeNe red laser (633 nm) for chloroplast auto‐fluorescence.

### Total gibberellic acid measurement

The sample leaves were harvested and stored at −80°C. The GA extraction was followed as the description with some modification (Baba *et al*., 2019). Approximately 100 mg leaves were ground by liquid N_2_ and incubated with 1 ml of 80% methanol solution containing 0.01% butylhydroxytoluene overnight at 4°C. After centrifugation at 11 758 ***g*** (Eppendorf centrifuge 5415D; Marshall Scientific, Hampton, NH, USA) for 10 min at 4°C, the supernatant was removed and mixed with hexane. The bottom layer was collected and dried by CentriVap Centrifugal Vacuum Concentrator (Labconco Corp., Kansas City, MO, USA). The dried pellet was resolved in 200 µl PBS for detection. To determine the concentration of GA, we followed the protocol of the enzyme‐linked immunosorbent assay kit (CEA759Ge; ELISA Kit for Gibberellic Acid (GA); Cloud Clone Corp., Katy, TX, USA). Approximately 50 µl of each sample was loaded into one well and incubated at 37°C after the regent was added. After the reaction, the signal was measured at 450 nm immediately by spectraMax M2 (Molecular Devices, San Jose, CA, USA). Three biological repeats were performed for total GA concentration measurement.

## Results

### An upregulated complementary DNA *ACGT8‐2* is a complementary DNA fragment of *NbDXR* gene

**Fig. 1:**
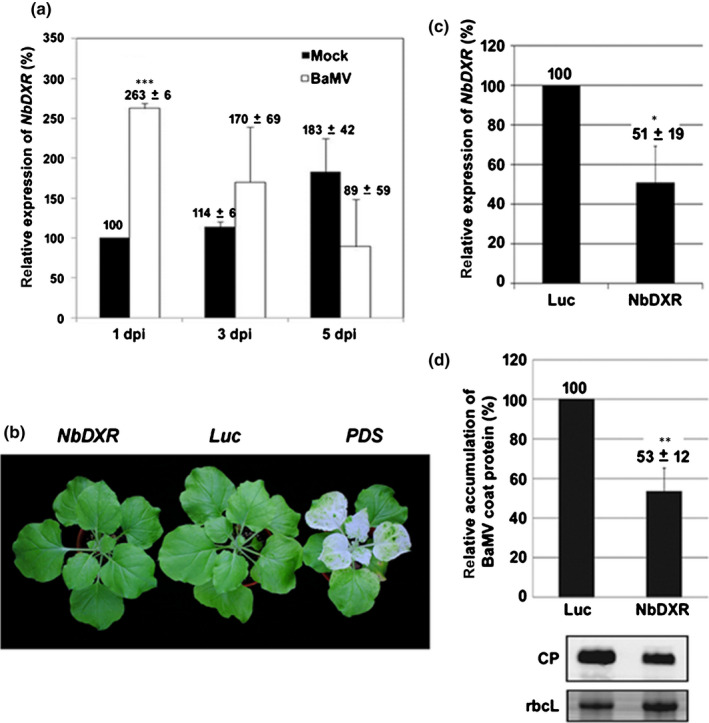
The expression profile of *NbDXR* and characterization of *NbDXR‐*knockdown *Nicotiana benthamiana* plants. DXR, 1‐deoxy‐d‐xylulose 5‐phosphate reductoisomerase. (a) The relative expression of *NbDXR* in mock and *Bamboo mosaic virus* (BaMV)‐inoculated leaves was measured by real‐time quantitative reverse transcription (qRT)‐PCR at 1, 3, and 5 d of post‐inoculation (dpi). The expression of the *actin* gene was used for normalization. (b) Morphological phenotype of control (*Luc*‐knockdown and *PDS*‐knockdown) and *NbDXR*‐knockdown plants. (c) Level of *NbDXR* in *Luc* and *NbDXR*‐knockdown leaves determined by real‐time qRT‐PCR. (d) Western blot analysis of viral BaMV coat protein (CP) accumulation. The level of rbcL was used for normalization. The accumulation in *Luc*‐knockdown plant (control) was set to 100%. Luc, *Luciferase*‐knockdown; PDS, *Phytoene desaturase*‐knockdown; NbDXR, *NbDXR*‐knockdown; rbcL, Rubisco large subunit (the loading control). Data are mean ± SE from at least three independent experiments. *, *P* < 0.05; **, *P* < 0.01; ***, *P* < 0.001 by Student’s *t*‐test.

The sequence of *ACGT8‐2*, a cDNA fragment identified by cDNA‐AFLP after BaMV inoculation in *N*. *benthamiana* (Cheng *et al*., 2010), showed 93% identity to tobacco DXR. Thus, *ACGT8‐2* could be part of the DXR orthologue in *N. benthamiana*. To clone the full‐length cDNA from *N. benthamiana*, we designed the primers for reverse transcription (RT)‐PCR based on the full‐length sequence of *NtDXR* (DQ839130) and the partial sequence of *NbDXR* from *N. benthamiana* (AM236596). We then compared the cloned full‐length cDNA sequence with the draft genome and transcriptome of *N. benthamiana* (Bombarely *et al*., 2012; Nakasugi *et al*., 2013). The sequence was the same as the predicted sequence of the nucleus‐encoded chloroplast *NbDXR* derived from the draft genome of *N. benthamiana*. On aligning NbDXR with other DXR orthologues, NbDXR shared 99% and 93% identity with tobacco DXR and tomato DXR, respectively (Fig. S1). Then, the upregulated expression profile of NbDXR after BaMV inoculation was confirmed by quantitative real‐time RT‐PCR (Fig. 1a). The result indicated that the expression of *NbDXR* increased to 2.6‐fold at 1 dpi. The expression declined gradually to that of the control level at 5 dpi.

### *NbDXR* is involved in BaMV accumulation

To examine the role of *NbDXR* in BaMV infection, we used VIGS to reduce the level of *NbDXR* in *N. benthamiana* plants. The phenotype of the *NbDXR*‐knockdown plants did not differ from that of *Luc* (luciferase)‐knockdown plants (Fig. 1b). The level of *NbDXR* was reduced to 51% of that of the control (Fig. 1c) and the level of BaMV CP in *NbDXR*‐knockdown plants was reduced to 53% of that of the control at 3 dpi (Fig. 1d).

**Fig. 2:**
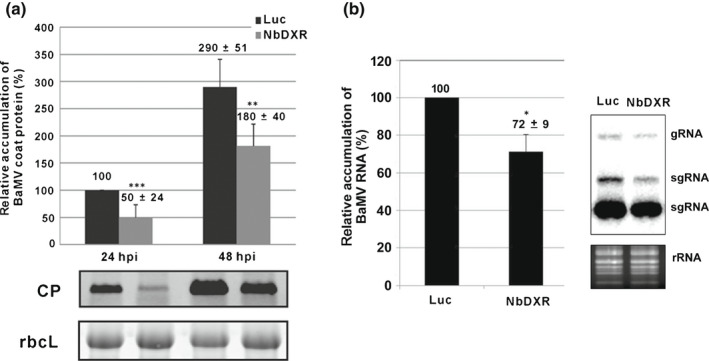
Accumulation of *Bamboo mosaic virus* (BaMV) in *NbDXR*‐knockdown protoplasts. Protoplasts were isolated from *Luc* or *NbDXR*‐knockdown *Nicotiana benthamiana* plants and inoculated with 500 ng BaMV viral RNA. Total protein and RNA were extracted from the inoculated protoplasts at 24 or 48 h post‐inoculation (hpi) and subjected to (a) Western blot analysis (24 and 48 hpi) and (b) Northern blot analysis (24 hpi). The accumulation of BaMV in *Luc*‐knockdown plant at 24 hpi was set to 100% for a comparison. DXR, 1‐deoxy‐d‐xylulose 5‐phosphate reductoisomerase; Luc, *Luciferase*‐knockdown; NbDXR, *NbDXR*‐knockdown; rbcL, Rubisco large subunit (the loading control); gRNA, guide RNA; rRNA, ribosomal RNA; sgRNA, single guide RNA. Data are mean ± SE from three independent experiments. *, *P* < 0.05; **, *P* < 0.01; ***, *P* < 0.001 by Student’s *t*‐test.

To further examine the effect of *NbDXR* knockdown on BaMV infection, we inoculated BaMV virion RNA into protoplasts from *NbDXR* and *Luc*‐knockdown plants. The results excluded the effect of cell‐to‐cell movement. The accumulation of BaMV CP was reduced to 50% and 62% of that of the control (*Luc*‐knockdown protoplasts) at 24 and 48 h post‐inoculation (hpi), respectively (Fig. 2a). The accumulation of viral RNA was also reduced to 72% of that of the control at 24 hpi (Fig. 2b). Hence, *NbDXR* could be involved in BaMV accumulation.

### NbDXR‐OFP is a chloroplast‐localized protein

Because DXR is a key enzyme of the MEP pathway that catalyzes DXP to MEP for terpenoid synthesis, DXR should be a chloroplast‐localized protein. To determine whether NbDXR is localized in chloroplasts, we constructed the fusion protein of NbDXR with the OFP at its C‐terminus (NbDXR‐OFP). After overexpression in *N. benthamiana*, confocal images showed that NbDXR‐OFP was localized in chloroplasts (Fig. S2). The result confirmed that NbDXR is a chloroplast‐localized protein.

### Overexpression of NbDXR increased BaMV accumulation

**Fig. 3:**
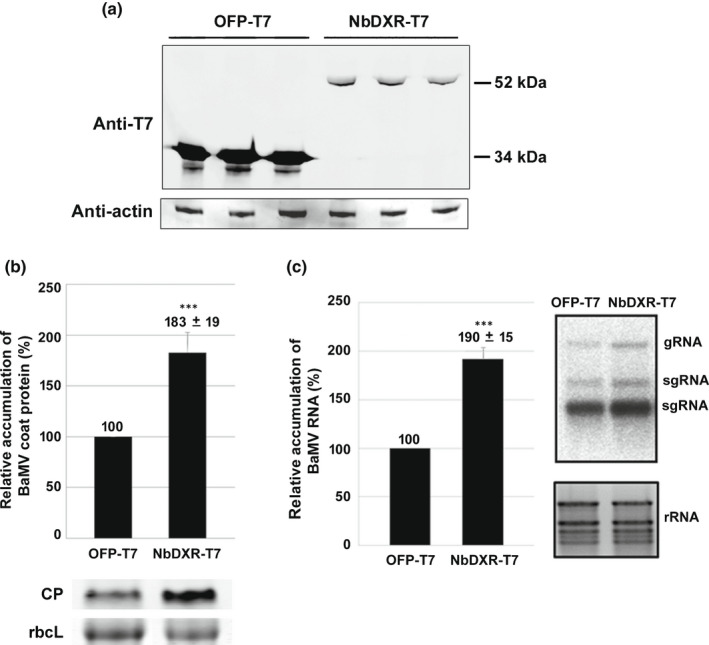
The accumulation of *Bamboo mosaic virus* (BaMV) coat protein (CP) in *Nicotiana benthamiana* with overexpression of NbDXR‐T7. (a) NbDXR‐T7 was transiently expressed in 1‐month‐old *N. benthamiana* for 3 d and subjected to Western blot analysis with antibody against T7‐tag. Then, leaves were inoculated with 1 µg BaMV virion on each leaf. Total protein and RNA were extracted from inoculated leaves at 3 d post‐inoculation and subjected to (b) Western blot analysis and (c) Northern blot analysis. (c) Data are the mean ± SE of at least three independent experiments. The accumulation of BaMV CP or RNA in OFP‐T7‐expressed leaves was set to 100%. DXR, 1‐deoxy‐d‐xylulose 5‐phosphate reductoisomerase; OFP, orange fluorescent protein; gRNA, guide RNA; rRNA, ribosomal RNA; sgRNA, single guide RNA. ***, *P* < 0.001 by Student’s *t*‐test.

The results of the *NbDXR*‐knockdown experiment suggested that BaMV accumulation involves NbDXR. We then transiently expressed NbDXR‐GFP in *N. benthamiana* leaves and inoculated BaMV onto NbDXR‐expressed leaves. Although we could detect the expressed protein on Western blot analysis (Fig. S3a), BaMV accumulation in NbDXR‐GFP‐expressed leaves was not significantly increased (Fig. S3b). We wondered whether NbDXR‐GFP with the C‐terminus 27 kDa green fluorescent protein (GFP) could have interfered with its function. We used the small peptide T7‐tag (11 amino acids) instead of GFP to fuse at the C‐terminus of NbDXR (NbDXR‐T7). NbDXR‐T7 was transiently expressed in *N. benthamiana* leaves inoculated with BaMV virion. We could detect the expressed protein on Western blot analysis (Fig. 3a). The BaMV CP and RNA levels in NbDXR‐T7‐expressed leaves were increased *c*. 1.8‐ and *c*. 1.9‐fold , respectively, compared with the control (OFP‐T7) (Fig. 3b,c). Thus, GFP fused at the C‐terminus of NbDXR affected its assistance in BaMV accumulation.

### DXR inhibitor fosmidomycin reduced the accumulation of BaMV

Because the fusion of GFP at the C‐terminus of DXR might alter the protein structure and fail to assist in BaMV accumulation, we wondered whether the catalytic activity of DXR is involved in BaMV accumulation. To test this hypothesis, we used the inhibitor fosmidomycin to block DXR activity. Fosmidomycin is used as a herbicide in plants and a drug against pathogenic bacteria and malaria parasites (Macreadie *et al*., 2000). We infiltrated plants with fosmidomycin at various concentrations and then inoculated BaMV virion on treated leaves. With 100 µM fosmidomycin, the accumulation of BaMV was decreased to < 60% of that of the control (Fig. S4). Therefore, the enzymatic activity of DXR could be involved in BaMV accumulation.

### The MEP pathway is involved in BaMV infection

**Fig. 4:**
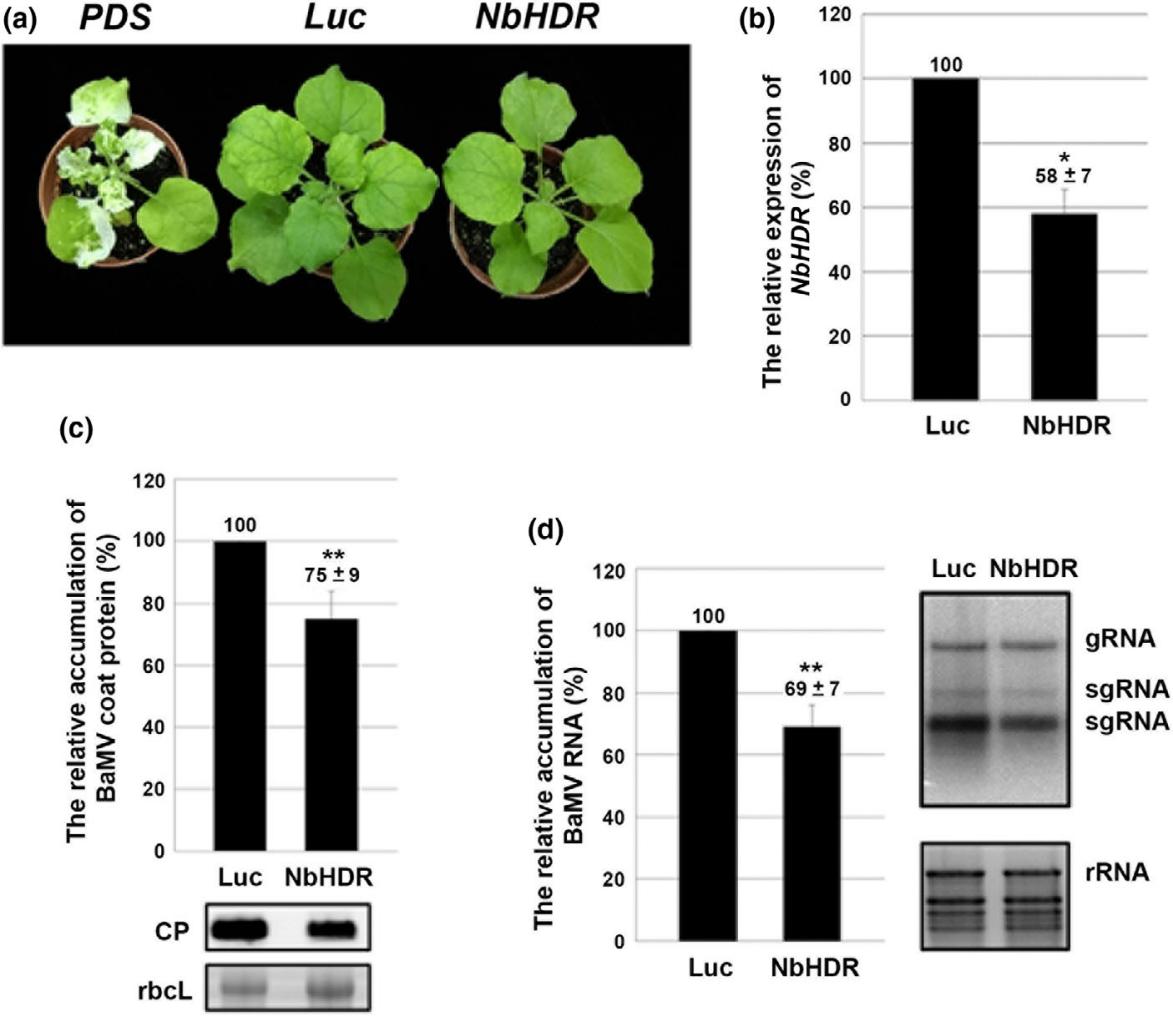
Characterization of *NbHDR‐*knockdown *Nicotiana benthamiana* plants. (a) Morphological phenotype of control (*Luc*‐knockdown and *PDS*‐knockdown) and *NbHDR*‐knockdown plants. (b) Expression of *NbHDR* in *Luc* and *NbHDR*‐knockdown leaves determined by real‐time quantitative reverse transcription PCR. Total protein and RNA were extracted from *Bamboo mosaic virus* (BaMV)‐inoculated knockdown leaves and subjected to (c) Western blot and (d) Northern blot analyses of viral accumulation. The level of rbcL was used for normalization for Western blot and rRNA for Northern blot analyses. The accumulation in *Luc*‐knockdown plants (control) was set to 100%. HDR, 1‐hydroxy‐2‐methyl‐butenyl 4‐diphosphate reductase; Luc, *Luciferase*‐knockdown; PDS, *Phytoene desaturase*‐knockdown; NbHDR, *NbHDR*‐knockdown; rbcL, Rubisco large subunit (the loading control); gRNA, guide RNA; rRNA, ribosomal RNA; sgRNA, single guide RNA. Data are mean ± SE from at least three independent experiments. *, *P* < 0.05; **, *P* < 0.01 by Student’s *t*‐test.

The results from knockdown and overexpression experiments with NbDXR suggested that NbDXR could be a host factor involved in BaMV accumulation. We wondered whether the protein factor NbDXR or the downstream metabolite was involved in BaMV infection. If one of the downstream metabolites is involved, the knockdown expression of downstream genes might also affect BaMV accumulation. We then selected two downstream genes of the MEP pathway, 4‐(cytidine 5′‐diphospho)‐2‐*C*‐methyl‐d‐erythritol kinase (*NbCMK*) and 1‐hydroxy‐2‐methyl‐2‐(*E*)‐butenyl‐4‐diphosphate reductase (*NbHDR*), for the knockdown experiments. The knocked down expression of *NbCMK* in *N. benthamiana* plants conferred an etiolated and dwarf phenotype (Fig. S5), but the knockdown of *NbHDR* conferred a normal phenotype, similar to that with mock treatment (Fig. 4a). Therefore, we used *NbHDR*‐knockdown plants for follow‐up experiments. The expression of *NbHDR* in knockdown plants was *c*. 58% of that of the control (Fig. 4b). The accumulation of BaMV CP and viral RNA in *NbHDR*‐knockdown plants was *c*. 75% and *c*. 69% of that of the control on Western blot (Fig. 4c) and Northern blot (Fig. 4d) analyses, respectively. The results suggested that NbHDR is also involved in BaMV accumulation. Overall, the results implied that the enzymes in the plastid MEP pathway are involved in supporting the accumulation of BaMV.

**Fig. 5:**
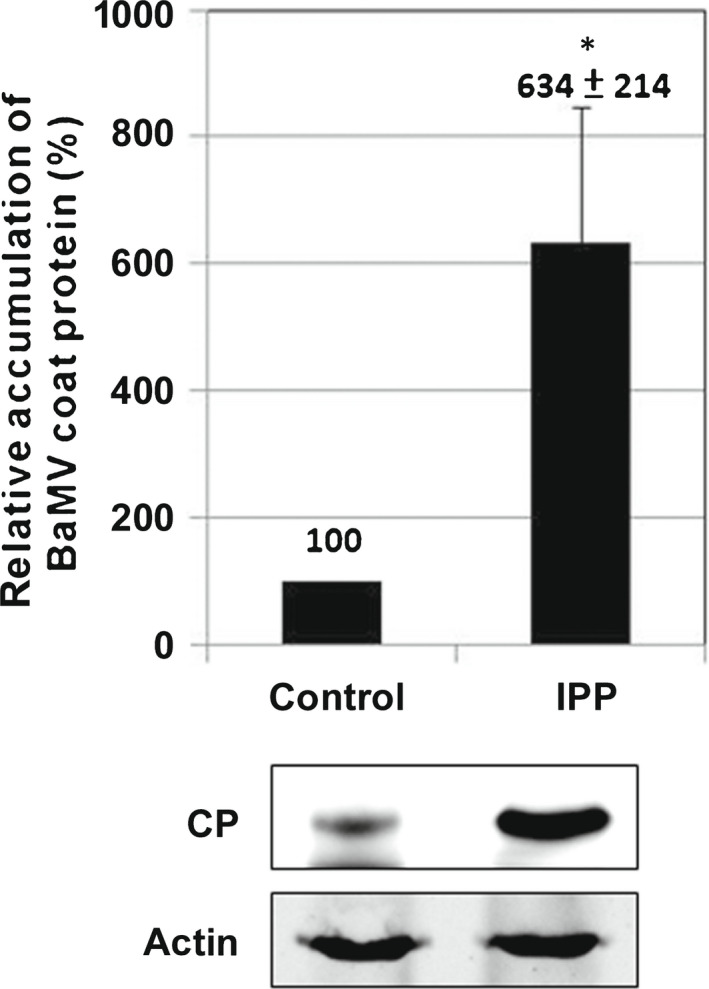
Accumulation of *Bamboo mosaic virus* (BaMV) coat protein (CP) in isopentenyl diphosphate (IPP)‐treated *Nicotiana benthamiana* protoplasts. Total protein was extracted from BaMV‐inoculated protoplasts and subjected to Western blot analysis of viral accumulation. The accumulation in protoplast incubation medium (control) was set to 100%. IPP: the incubation medium containing 150 μM IPP; actin: protein used as the loading control for Western blot analysis. Data are mean ± SE from at least three independent experiments. *, *P* < 0.05 by Student’s *t*‐test.

The main products of the MEP pathway are interchangeable IPP and dimethylallyl pyrophosphate (DMAPP). These metabolites could be further modified to produce geranylgeranyl monophosphate and GGPP. To reveal how important the chemical IPP is in BaMV accumulation, we provided IPP in the incubation medium of BaMV‐inoculated protoplasts. The addition of IPP could increase BaMV accumulation to six‐fold that of control protoplasts (Fig. 5). This result led to another question of whether IPP or the downstream metabolites had the effect.

### Gibberellin could be the final product derived from the MEP pathway involved in BaMV accumulation

**Fig. 6:**
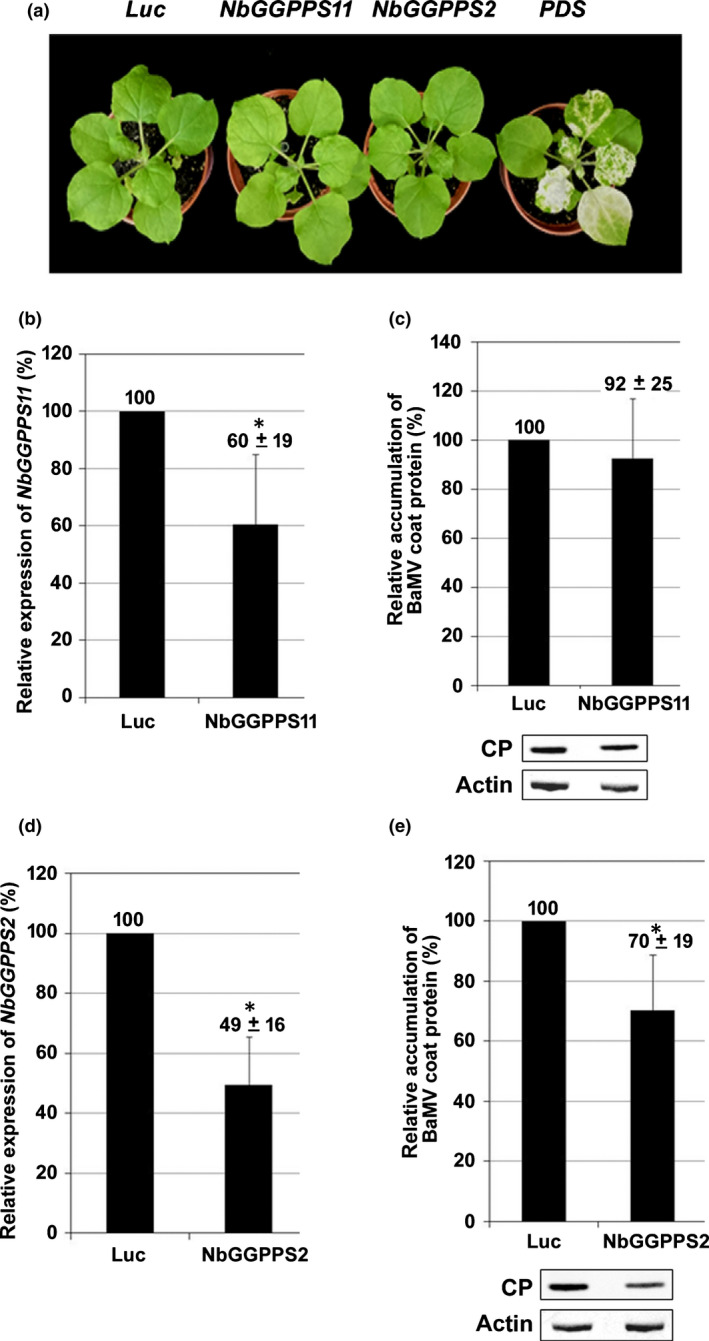
Characterization of *NbGGPPS11‐* and *NbGGPPS2‐*knockdown *Nicotiana benthamiana* plants. (a) Morphological phenotype of control (*Luc* and *PDS*‐knockdown) and *NbGGPPS11* and *NbGGPPS2‐*knockdown plants. The expression of (b) *NbGGPPS11* and (d) *NbGGPPS2* in *NbGGPPS11* and *NbGGPPS2*‐knockdown leaves, respectively, and control leaves was determined by real‐time quantitative reverse transcription PCR. Total protein was extracted from *Bamboo mosaic virus* (BaMV)‐inoculated (c) *NbGGPPS11* and (e) *NbGGPPS2*‐knockdown leaves and subjected to Western blot analysis of viral accumulation. Actin level was used for normalization. The gene expression or viral accumulation in *Luc*‐knockdown plants (control) was set to 100%. CP, coat protein; GGPPS, geranylgeranyl pyrophosphate synthase; Luc, *Luciferase*‐knockdown; PDS, *Phytoene desaturase*‐knockdown; NbGGPPS11, *NbGGPPS11*‐knockdown; NbGGPPS2, *NbGGPPS2*‐knockdown. Data are mean ± SE from at least three independent experiments. *, *P* < 0.05 by Student’s *t*‐test.

To examine the downstream genes using the final products of the MEP pathway (IPP/DMAPP) for involvement in BaMV accumulation, we examined the genes for GGPP synthesis. Because GGPP is the most critical metabolite in the plastid as the precursor for isoprenoid synthesis, we examined the importance of GGPP in BaMV accumulation. From the study of isoprenoid synthesis in *Arabidopsis*, GGPPS2 and GGPPS11 are two of the 12 isoforms expressed in green tissue to synthesize GGPP (Ruiz‐Sola *et al*., 2016). We then searched for the *GGPPS*s gene in the *N*. *benthamiana* database. The polypeptide designated as NbGGPP2 (Nbv6.1trP5493) was shown to have 99.4% and 64.8% identity with that of *N. tabacum* (NtGGPPS2, AHA58682.1) and *Arabidopsis* (AtGGPPS2, NP_179960.1), respectively. The amino acid sequence of NbGGPPS11 (BBF45635.1) was shown to have 98.4% and 67.8% identity with that of *N. tabacum* (NtGGPPS11, AHA58681.1) and *Arabidopsis* (AtGGPPS2, NP_195399.1), respectively. We then knocked down the expression of NbGGPPS11 and NbGGPPS2 from *N. benthamiana*. We did not observe any obvious morphological change in knockdown plants (Fig. 6a). The expression of *NbGGPPS11* and *NbGGPPS2* was 60% and 40% of that of control leaves, respectively (Fig. 6b,d). The accumulation of BaMV did not differ between *NbGGPPS11*‐knockdown leaves and control leaves (Fig. 6c), but the accumulation of BaMV CP in *NbGGPPS2*‐knockdown leaves was significantly reduced to 70% (Fig. 6e). Thus, GGPP synthesized by NbGGPPS11 or NbGGPPS2 might have different roles.

From study of the gene co‐expression network (GCN) in *Arabidopsis*, GGPPS11 is involved in the major production of plastid isoprenoids. Furthermore, GGPPS11 was found to physically interact with the enzymes that use GGPP for producing carotenoids, Chls, tocopherols, phylloquinone, and plastoquinone (Ruiz‐Sola *et al*., 2016). By contrast, the minor expressed GGPPS2 was linked to GA synthesis on GCN analysis (Ruiz‐Sola *et al*., 2016). Accordingly, the accumulation of BaMV might be linked with NbGGPPS2‐related GA synthesis rather than enzyme(s) or product(s) involved in NbGGPPS11‐related photosynthesis.

**Fig. 7:**
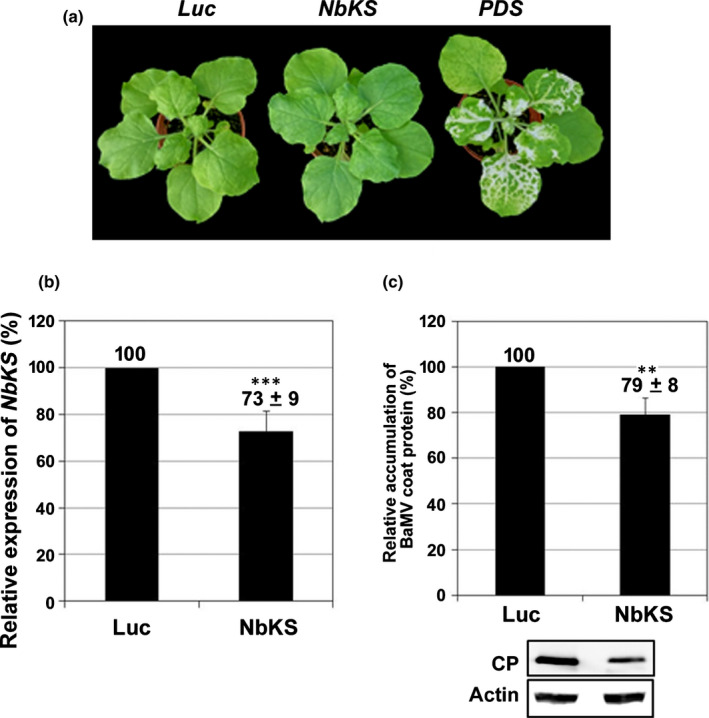
Characterization of *NbKS‐*knockdown *Nicotiana benthamiana* plants. (a) Morphological phenotype of control (*Luc*‐knockdown and *PDS*‐knockdown) and *NbKS*‐knockdown plants. (b) The expression of *NbKS* in *Luc*‐ and *NbKS*‐knockdown leaves determined by real‐time quantitative reverse transcription PCR. (c) Western blot analysis of viral *Bamboo mosaic virus* (BaMV) coat protein (CP) accumulation. Actin level was used for normalization. The gene expression or viral accumulation level in *Luc*‐knockdown plants (control) was set to 100%. KS, *ent*‐kaurene synthase; Luc, *Luciferase*‐knockdown; PDS, *Phytoene desaturase*‐knockdown; NbKS, *NbKS*‐knockdown. Data are mean ± SE from at least three independent experiments. **, *P* < 0.01; ***, *P* < 0.001 by Student’s *t*‐test.

To validate that the GA synthesis pathway is involved in BaMV accumulation, we knocked down the expression of KS to block GA synthesis but found no obvious effect on plant growth in *NbKS*‐knockdown plants (Fig. 7a). The mRNA expression of *NbKS* was reduced to 73% in knockdown leaves compared with the control (Fig. 7b), and the accumulation of BaMV CP was significantly decreased to 79% of that of the control (Fig. 7c). These results confirm that the GA synthesis pathway was involved in the accumulation of BaMV.

**Fig. 8:**
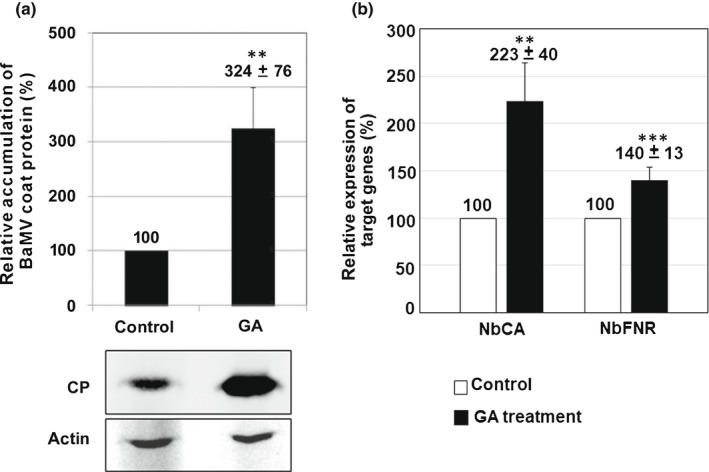
Characterization of gibberellic acid (GA)‐treated *Nicotiana benthamiana* protoplasts. (a) Relative accumulation of *Bamboo mosaic virus* (BaMV) coat protein (CP) in GA‐treated protoplasts. Total protein was extracted from BaMV‐inoculated protoplasts and subjected to Western blot analysis of viral accumulation. The accumulation in protoplasts with normal incubation medium as the control was set to 100%. GA: the incubation medium containing 150 μM GA; actin: protein on Western blot used as the loading control. (b) Relative messenger RNA expression of NbCA and NbFNR in protoplasts after GA treatment. Total RNA was extracted from protoplasts and subjected to real‐time reverse transcription PCR. CA, carbonic anhydrase; FNR, ferredoxin NADP^+^ oxidoreductase. Data are mean ± SE from at least three independent experiments. **, *P* < 0.01; ***, *P* < 0.001 by Student’s *t*‐test.

**Fig. 9:**
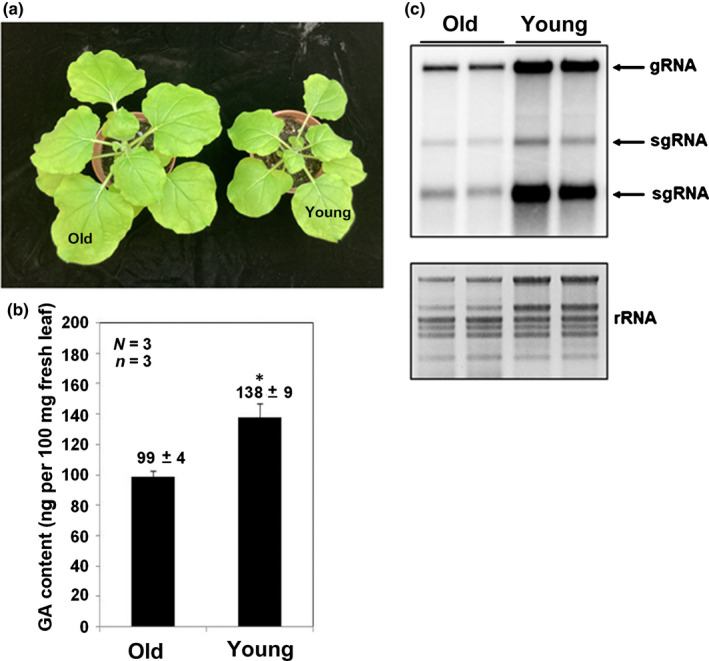
The gibberellic acid (GA) content and the accumulation of *Bamboo mosaic virus* (BaMV) RNA in the old and young leaves of *Nicotiana benthamiana*. (a) The leaves chosen for the experiment from old and young plants are indicated. (b) The GA content from the leaf of the old and young plants was determined by a competitive GA enzyme‐linked immunosorbent assay. Data above bars are mean ± SE from three independent experiments (*N* = 3) with three plants in each experiment (*n* = 3). *, *P* < 0.05 by Student’s *t*‐test. (c) Total RNA was extracted from BaMV‐inoculated protoplasts derived from the chosen leaf and subjected to Northern blot analysis. gRNA, guide RNA; rRNA, ribosomal RNA; sgRNA, single guide RNA.

To confirm that GA is the assistance factor critical for BaMV accumulation, we added GA in the incubation medium of protoplasts after BaMV inoculation. The accumulation of BaMV was increased to 3.24‐fold of that of the control (Fig. 8a). Thus, GA could be the critical factor involved in BaMV accumulation. The crucial precursor of GA is *ent*‐kaurene synthesized from GGPP and is known to occur in the stroma of proplastids or developing chloroplasts but not in mature chloroplasts (Aach *et al*., 1995, 1997). Therefore, the endogenous GA level could be higher in younger expanding leaves than in older expanded mature leaves (Fig. 9). We then examined the accumulation of BaMV in the same position leaf at different ages. BaMV RNA was more efficiently accumulated in young leaves than old leaves (Fig. 9c).

Because GA is involved in many developmental processes *in planta*, such as leaf expansion, the expression of development‐related genes should be turned on. These GA‐induced expressing genes could be involved in BaMV accumulation (Huang *et al*., 2017a). We then examined the expression of some known host factors, such as ferredoxin NADP^+^ oxidoreductase (FNR) (Chen *et al*., 2022) and carbonic anhydrase (CA) (Chen *et al*., 2017c). The expression of NbCA and NbFNR in GA‐treated leaves was elevated to 2.23‐fold and 1.4‐fold, respectively, compared with untreated leaves (Fig. 8b). Hence, the production of GA via the MEP pathway in chloroplasts could upregulate the expression of nuclear‐encoded chloroplast proteins including CA and FNR that were revealed to assist in BaMV accumulation.

## Discussion

Combining screening of differentially expressed genes and knockdown/overexpression experiments is a successful strategy to identify the host genes involved in virus infection (Cheng *et al*., 2010). We have used this strategy and identified several genes supporting or impairing BaMV infection (Huang *et al*., 2017a). NbDXR was one of the differentially expressed genes screened by cDNA‐AFLP (Cheng *et al*., 2010). The results of knockdown (loss of function; Figs 1, 2) and overexpression (gain of function; Fig. 3) suggested that NbDXR is involved in BaMV RNA accumulation. The expression of NbDXR‐T7 (Fig. 3) or NbDXR‐GFP (Fig. S3) in *N. benthamiana* had a different effect on the accumulation of BaMV. Thus, adding an additional bulky structure such as GFP (27 kDa) to the C‐terminus of NbDXR might block the proper folding of NbDXR or the interaction with the substrate compared with adding the T7‐tag, the short oligopeptide. Furthermore, the accumulation of BaMV decreased when plants were infiltrated with the DXR inhibitor fosmidomycin (Fig. S4). These results indicate that the proper structure with the enzymatic activity of NbDXR is critical for BaMV accumulation.

To trace the vital components involved in BaMV replication, we demonstrated that a crucial metabolite of the MEP pathway, IPP, could increase BaMV accumulation (Fig. 5). IPP is the precursor for monoterpenes, Chls, tocopherols, plastoquinone, phylloquinone, carotenoids, abscisic acid, and GA synthesis (Pulido *et al*., 2013). Downstream of IPP is the intermediate metabolite GGPP synthesized by GGPPS. There are 12 paralogous *GGPPS* genes (*GGPPS1*–*GGPPS12*) in the genome of *Arabidopsis thaliana* (Lange & Ghassemian, 2003). However, excluding those expressed in the cytoplasm and pseudogene, seven are the plastid enzymes (GGPPS2, 6, 7, 8, 9, 10, and ‐11). GGPPS11 and GGPPS2 are the only two isoforms expressed in the plastid of green tissue (Lange & Ghassemian, 2003). *GGPPS11* is the major expressed gene required for all major groups of isoprenoid synthesis and is suggested to be vital for plant development (Ruiz‐Sola *et al*., 2016; Hedden, 2020). By contrast, *GGPPS2* is a less‐expressed gene and after GGPP synthesis was mainly related to GA synthesis. Each GGPPS may interact with GGPP‐consuming enzymes and channel the newly synthesized GGPP for producing downstream isoprenoids (Ruiz‐Sola *et al*., 2016). The relationship between the MEP pathway and downstream GA synthesis was confirmed by the measurement of GA content in the knockdown plants. The genes involved in GA syntheses, like *NbDXR* and *NbKS*, showed a reduced level of GA when knocked down but not those of unrelated genes did not (Fig. S6).

Because GA production occurs in the stroma of proplastids or developing chloroplasts, young leaves could produce more GA (Aach *et al*., 1995, 1997). We demonstrated that young leaves had more GA production (Fig. 9), which could be involved in more efficient accumulation of BaMV (Fig. 9c). This is another line of evidence that GA could support the accumulation of BaMV.

Although we revealed that GA is an assistance factor for BaMV accumulation, how GA triggers the assisting role is still not known. GA is a vital factor for most major developmental processes, including seed germination, stem elongation, leaf expansion, and flowering by stimulating cell division and elongation (Hedden & Sponsel, 2015; Teotia & Tang, 2015). To execute these processes, bioactive GA interacts with its receptor GIBBERELLIN INSENSITIVE DWARF 1 (GID1) that induces the conformational change of the GA–GID1 complex and binds to the DELLA domain of DELLAs (Griffiths *et al*., 2006; Willige *et al*., 2007; Murase *et al*., 2008). The complex GA–GID1–DELLA then binds to the E3 ubiquitin ligase, which leads to the degradation of DELLAs by 26S proteasome (Dill *et al*., 2004). Because DELLAs are growth‐suppressing proteins of the transcriptional regulators, the removal of these suppressors by GA activation could turn on the downstream genes (Sun & Gubler, 2004). Whether the genes turned on by GA signaling, such as those for leaf expansion, are the crucial factors for BaMV accumulation is not known. However, we showed here that GA could upregulate the expression of nuclear‐encoded chloroplast genes such as CA and FNR, which could assist in BaMV accumulation.

**Fig. 10:**
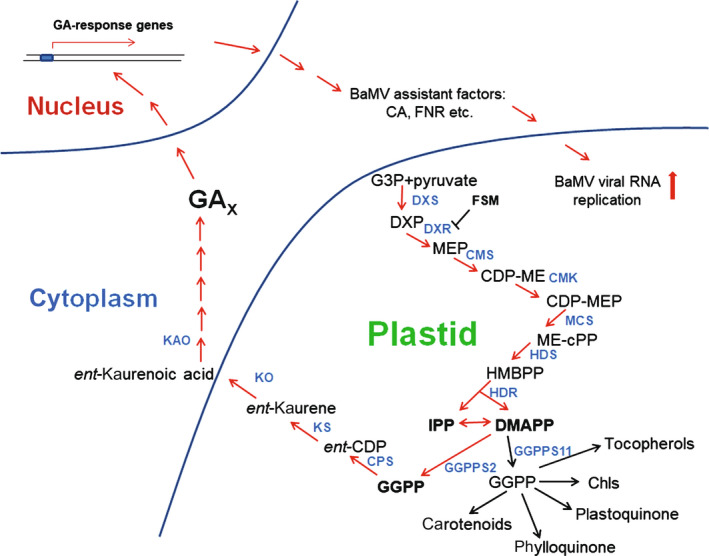
A schematic representation of the chloroplast MEP pathway involved in *Bamboo mosaic virus* (BaMV) accumulation. The product IPP of the MEP pathway was converted to GGPP by GGPPS2 and then via the gibberellic acid (GA) synthesis pathway to synthesize GA. GA could upregulate GA‐response genes that might include BaMV accumulation‐assistance factors, such as CA and FNR. The arrows are the metabolic steps in the MEP pathway and their downstream products, including the GA biosynthesis. The arrows in red are the steps involved in BaMV accumulation. The blunt‐ended arrow represents the enzyme inhibitor. FSM, fosmidomycin. Enzymes: DXS, 1‐deoxy‐d‐xylulose‐5‐phosphate synthase; DXR, 1‐deoxy‐d‐xylulose 5‐phosphate reductoisomerase; CMS, 4‐diphosphocytidyl‐2‐*C*‐methyl‐d‐erythritol synthase; CMK, 4‐diphosphocytidyl‐2‐*C*‐methylerythritol kinase; MCS, 2‐*C*‐methyl‐d‐erythritol 2,4‐cyclodiphosphate synthase; HDS, 4‐hydroxy‐3‐methylbut‐2‐en‐1‐yl diphosphate synthase; HDR, 1‐hydroxy‐2‐methyl‐butenyl 4‐diphosphate reductase; GGPPS2, geranylgeranyl pyrophosphate synthase 2; GGPPS11, geranylgeranyl pyrophosphate synthase 11; CPS, *ent*‐copalys diphosphate synthase; KS, *ent*‐kaurene synthase; KO, *ent*‐kaurene oxidase; KAO, *ent*‐kaurenoic acid oxidase; CA, carbonic anhydrase; FNR, ferredoxin‐NADP^+^ oxidoreductase. Metabolites: G3P, d‐glyceraldehyde 3‐phosphate; DXP, 1‐deoxy‐d‐xylulose 5‐phosphate; MEP, 2‐*C*‐methyl‐d‐erythritol 4‐phosphate; CDP‐ME, 4‐diphosphocytidyl‐2‐*C*‐methyl‐d‐erythritol; CDP‐MEP, 2‐phospho‐4‐diphosphocytidyl‐2‐*C*‐methyl‐d‐erythritol; ME‐cPP, 2‐*C*‐methyl‐d‐erythritol 2,4‐cyclodiphosphate; HMBPP, 1‐hydroxy‐2‐methyl‐2‐(*E*)‐butenyl 4‐diphosphate. IPP, isopentenyl diphosphate; DMAPP, dimethylallyl pyrophosphate; GGPP, geranylgeranyl pyrophosphate; *ent*‐CDP, *ent*‐copalys diphosphate.

From the results of this study, we propose a model (Fig. 10) that the upregulated expression of DXR immediately after BaMV infection (Fig. 1) could trigger the metabolic process of the MEP pathway in chloroplasts. The products of the MEP pathway involved the specific enzyme GGPPS2, which synthesizes GGPP used for GA biosynthesis. The results were also confirmed by the measurement of GA content in the BaMV‐inoculated plant, where the GA content was elevated at 1 dpi and then declined (Fig. S7). The newly synthesized GA could turn on the GA‐response genes that might include the factors such as CA and FNR to assist in BaMV accumulation in chloroplasts. Finally, the elevated level of GA could then turn down the expression level of NbDXR (Fig. S8) as the feedback effect.

## Author contributions

Y‐PH, I‐HC and Y‐SK performed the experiments; Y‐PH, Y‐HH and C‐HT took part in data analysis; Y‐PH and C‐HT designated the research and wrote the article.

## Supporting information


**Fig. S1** The amino acid sequence alignment of DXRs.
**Fig. S2** Localization of NbDXR‐OFP in protoplasts of *Nicotiana benthamiana* by confocal microscopy.
**Fig. S3** The accumulation of BaMV in *Nicotiana benthamiana* plants with overexpression of NbDXR‐GFP.
**Fig. S4** The effect of fosmidomycin on the accumulation of BaMV.
**Fig. S5** The morphological phenotype of control (*Luc* and *Phytoene desaturase*‐knockdown) and *NbCMK‐*knockdown plants.
**Fig. S6** The GA content in the knockdown plants.
**Fig. S7** The GA content in the BaMV‐inoculated plants.
**Fig. S8** The relative expression levels of *NbDXR* after GA treatment.

**Table S1** List of primer names, sequences, and purpose in this study.
Please note: Wiley Blackwell are not responsible for the content or functionality of any Supporting Information supplied by the authors. Any queries (other than missing material) should be directed to the *New Phytologist* Central Office.

## Data Availability

The data supporting the findings of this study are available from the corresponding author (C‐HT) upon request.
