# Supplementary material for: The gibberellic acid derived from the plastidial MEP pathway is involved in the accumulation of Bamboo mosaic virus
Source: New Phytol. 2022 Jun 22;235(4):1543–57. doi: 10.1111/nph.18210 (PMC9543464; doi:10.1111/nph.18210)
Supplement: Supplementary file 1 — Fig. S1 The amino acid sequence alignment of DXRs. Fig. S2 Localization of NbDXR‐OFP in protoplasts of Nicotiana benthamiana by confocal microscopy. Fig. S3 The accumulation of BaMV in Nicotiana benthamiana plants with overexpression of NbDXR‐GFP. Fig. S4 The effect of fosmidomycin on the accumulation of BaMV. Fig. S5 The morphological phenotype of control (Luc and Phytoene desaturase‐knockdown) and NbCMK‐knockdown plants. Fig. S6 The GA content in the knockdown plants. Fig. S7 The GA content in the BaMV‐inoculated plants. Fig. S8 The relative expression levels of NbDXR after GA treatment. [file NPH-235-1543-s001.pdf]

New Phytologist **Supporting Information**

Article title: **The gibberellic acid derived from the plastidial MEP pathway is involved in the accumulation of *Bamboo mosaic virus***

Authors: Ying-Ping Huang, I-Hsuan Chen, Yu-Shun Kao, Yau-Heiu Hsu and Ching-Hsiu Tsai

Fig. S1 The amino acid sequence alignment of DXRs.

Fig. S2 Localization of NbDXR-OFP in protoplasts of *N. benthamiana* by confocal microscopy.

Fig. S3 The accumulation of BaMV in *N. benthamiana* plants with overexpression of NbDXR-GFP.

Fig. S4 The effect of fosmidomycin on the accumulation of BaMV.

Fig. S5 The morphological phenotype of control (*Luc*- and *PDS*-knockdown) and *NbCMK*-knockdown plants.

Fig. S6 The GA content in the knockdown plants.

Fig. S7 The GA content in the BaMV-inoculated plants.

Fig. S8 The relative expression levels of *NbDXR* after GA treatment.

```
       1        10        20        30        40        50        60
NbDXR  MALNLLSPSEIKTISFLDTSKSSYNLNHLKFQGGVAIKRKEWSGISGKRVQCSVQAPPP-
NtDXR  MALNLLSPSEIKTISFLDTSTSSYNLNHLKFQGGVAIKRKEWSGISGKRVQCSVQAPPP-
SlDXR  MALNLLSPAEIKSISFLDNSKSSYNLSHLKFTGGLSIRRKECSGAFAKRVQCSAQPPPPP

       61       70        80        90        100       110      120
NbDXR  AWPGRAVAEA-RKTWDGPKPISIVGSTGSIGTQTLDIVAENPDKFRVVALAAGSNVTLLA
NtDXR  AWPGRAVAEA-RKTWDGPKPISIVGSTGSIGTQTLDIVAENPDKFRVVALAAGSNVTLLA
SlDXR  AWPGRAVAEPGRQSWDGPKPISIVGSTGSIGTQTLDIVAENPDKFRVVALAAGSNVTLLA

       121      130       140       150       160       170      180
NbDXR  DQVKTFRPKLVAVRNESLVEELKDALADMEDKPEIIPGEQGIIEVARHPDAVTVVTGIVG
NtDXR  DQVKTFKPKLVAVRNESLVEELKDALADMEDKPEIIPGEQGIIEVARHPDAVTVVTGIVG
SlDXR  DQVKTFRPKLVAVRNESLVEELKDALADMEDKPEIIPGEQGVIEVARHPDAVTVVTGIVG

       181      190       200       210       220       230      240
NbDXR  CAGLKPTVAAIEAGKDIALANKETLIAGGPFVLPLAHKHKVKILPADSEHSAIFQCIQGL
NtDXR  CAGLKPTVAAIKAGKDIALANKETLIAGGPFVLPLAHKHKVKILPADSEHSAIFQCIQGL
SlDXR  CAGLKPTVAAIEAGKDIALANKETLIAGGPFVLPLAHKHKVKILPADSEHSAIFQCIQGL

       241      250       260       270       280       290      300
NbDXR  PEGALRRIILTASGGAFRDLPVEKLKEVKVADALKHPNWNMGKKITVDSATLFNKGLEVI
NtDXR  PEGALRRIILTASGGAFRDLPVEKLKEVKVADALKHPNWNMGKKITVDSATLFNKGLEVI
SlDXR  PEGALRRIILTASGGAFRDWPVEKLKEVKVADALKHPNWNMGKKITVDSATLFNKGLEVI

       301      310       320       330       340       350      360
NbDXR  EAHYLFGAEYDDIEIVIHPQSIIHSMVETQDSSVLAQLGWPDMRLPILYTLSWPDRIYCS
NtDXR  EAHYLFGAEYDDIEIVIHPQSIIHSMVETQDSSVLAQLGWPDMRLPILYTLSWPDRIYCS
SlDXR  EAHYLFGAEYDNIEIVIHPQSIIHSMVETQDSSVLAQLGWPDMRLPILYTLSWPDRVYCS

       361      370       380       390       400       410      420
NbDXR  EVTWPRLDLCKLGSLTFKVPDNVKYPSMDLAYAAGRAGGTMTGVLSAANEMAVELFISER
NtDXR  EVTWPRLDLCKLGSLTFKAPDNVKYPSMDLAYAAGRAGGTMTGVLSAANEKAVELFISER
SlDXR  EITWPRLDLCKLGSLTFKAPDNVKYPSMDLAYSAGRAGGTMTGVLSAANEKAVELFISER
```

Fig. S1 The amino acid sequence alignment of DXRs. The sequences from *Nicotiana benthamiana* (NbDXR), *N. tabacum* (NtDXR, Accession: NP_001312964.1), and *Solanum lycopersicum* (SlDXR, Accession: AAK96063.2) were aligned.

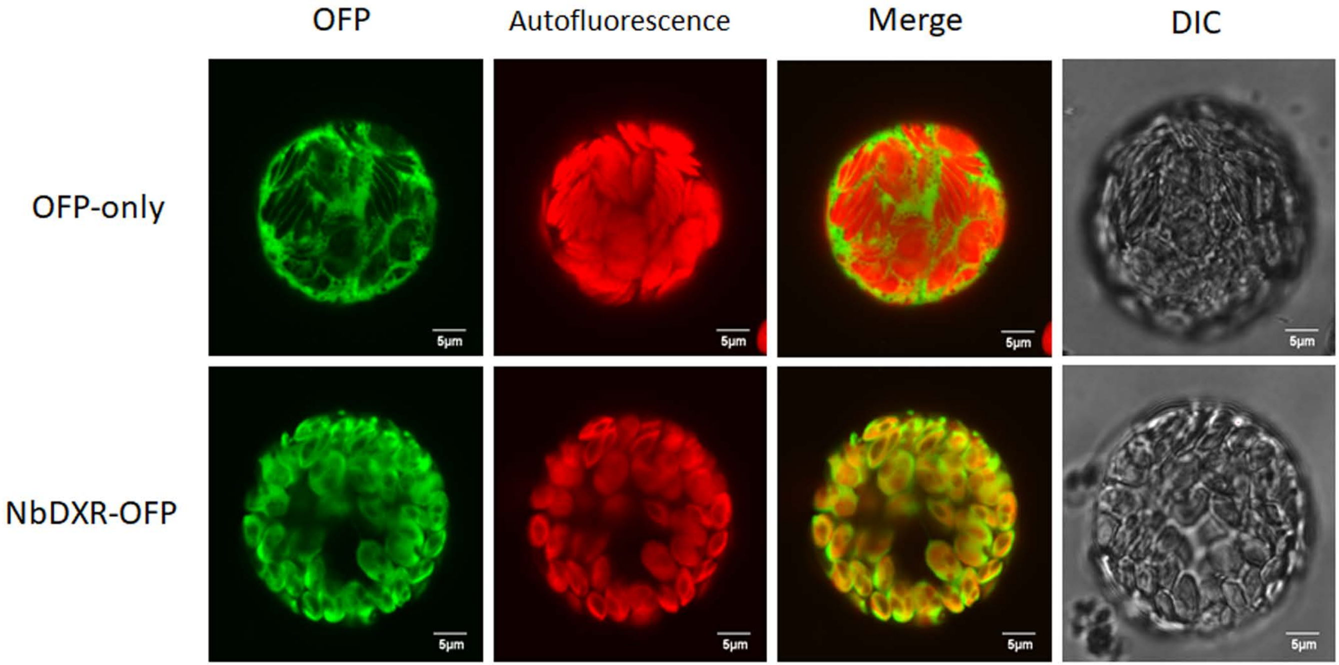

Fig. S2 Localization of NbDXR-OFP in protoplasts of *N. benthamiana* by confocal microscopy. OP and NbDXR-OFP were transiently expressed by agro-infiltration in *N. benthamiana* leaves. Protoplasts were isolated from post-infiltrated leaves at 3 days and examined by confocal microscopy. A merged image shows NbDXR-OFP in green and chloroplasts with auto-fluorescent labeled in red. Images were obtained by using the Olympus Fluoview FV1000 confocal microscope with 543 nm and 633 nm laser excitations. Scale bar is 5 μm.

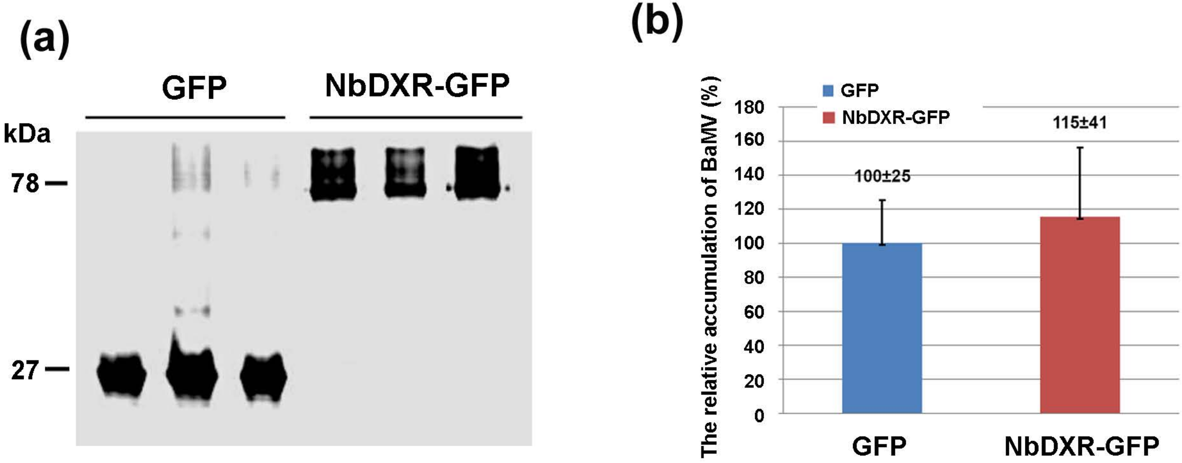

Fig. S3 The accumulation of BaMV in *N. benthamiana* plants with overexpression of NbDXR-GFP. **(a)** GFP and NbDXR-GFP were transiently expressed in *N. benthamiana* plants by agro-infiltration and analyzed by western blot with anti-GFP antiserum. **(b)** One-month-old *N. benthamiana* plants were agro-infiltrated to transiently express GFP or NbDXR-GFP for 3 days and inoculated with 1 μg BaMV virion. Total proteins were extracted from inoculated leaves at 3 days post-inoculation, and subjected to western blot analysis. Data are mean±SE of three independent experiments. The accumulation of BaMV CP in GFP-knockdown plants was set to 100% for comparison.

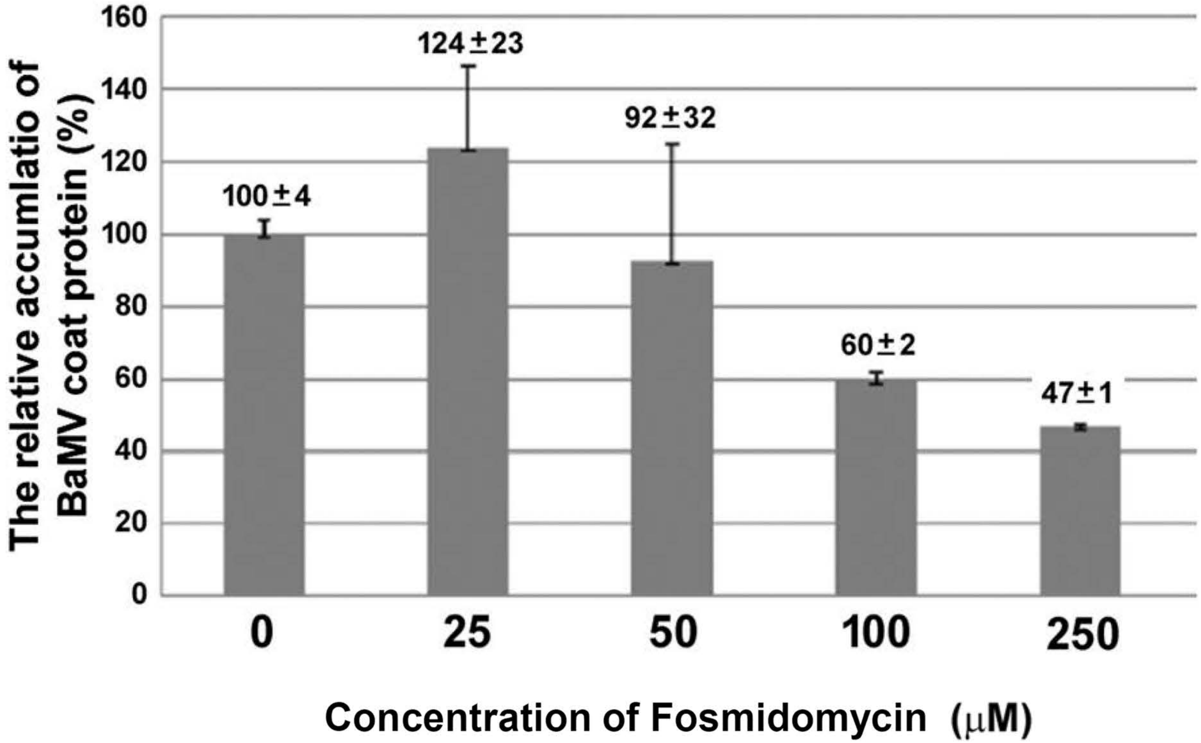

Fig. S4 The effect of fosmidomycin on the accumulation of BaMV. Accumulation of BaMV CP on fosmidomycin-treated *N. benthamiana* plants. Total protein was extracted from inoculated leaves at 3 days post inoculation (dpi) and subjected to western blot analysis. Data are mean±SE of three independent experiments. The accumulation of BaMV CP in control plants (0 μM fosmidomycin treatment) was set to 100% for comparison.

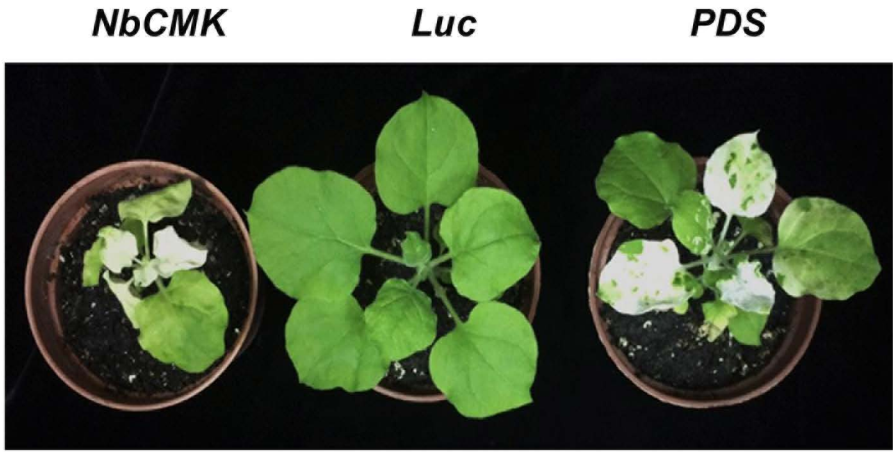

Fig. S5 The morphological phenotype of control (*Luc*- and *PDS*-knockdown) and *NbCMK*-knockdown plants.

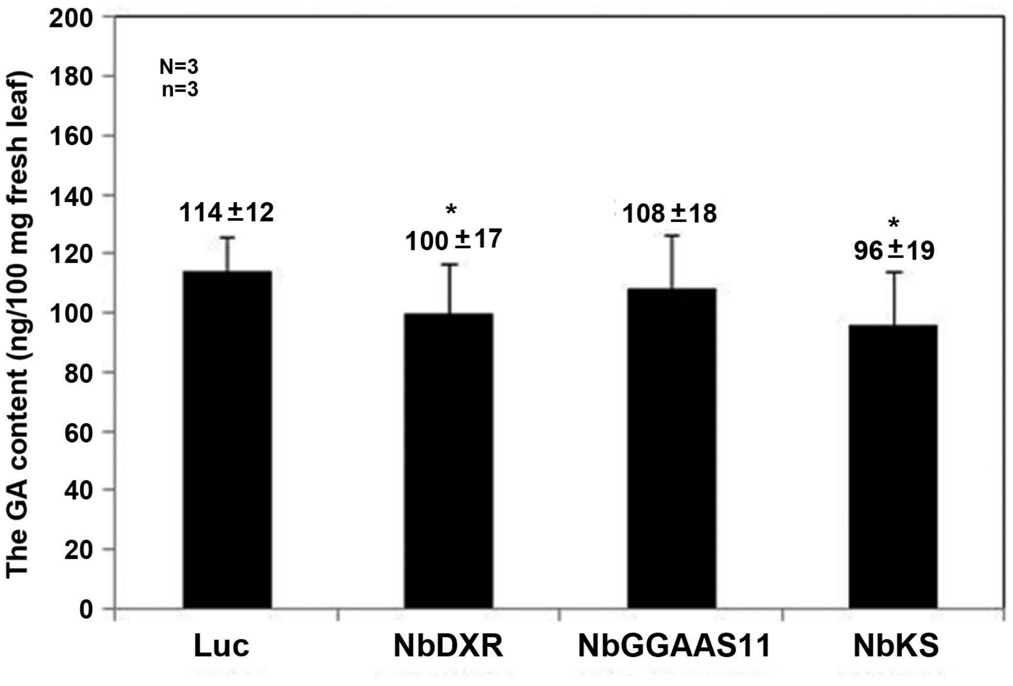

Fig. S6 The GA content in the knockdown plants. The GA content from the leaf of the knockdown plants indicated was determined by a competitive GA ELISA assay. Data above bars are mean±SE from three independent experiments (N=3) with three plants in each experiment (n=3). *$p < 0.05$ by Student *t*-test.

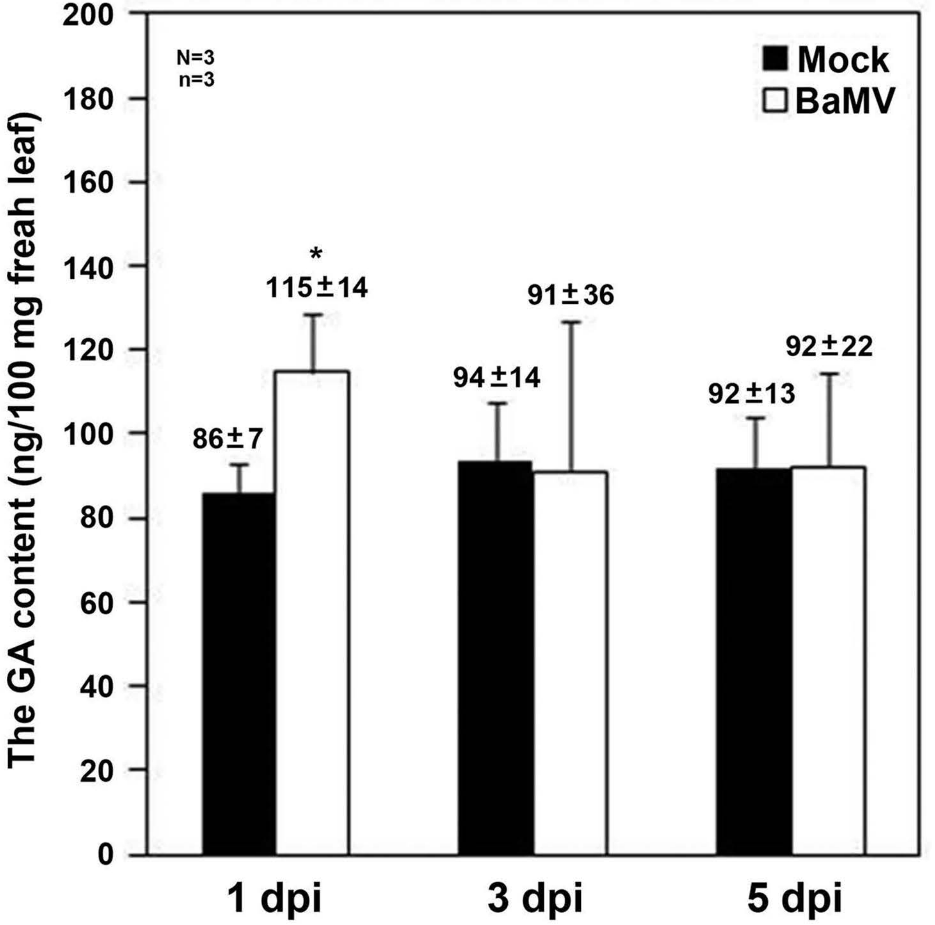

Fig. S7 The GA content in the BaMV-inoculated plants. The GA content from the leaf of the BaMV-inoculated plants at 1, 3, and 5 days of post-inoculation (dpi) was determined by a competitive GA ELISA assay. Data above bars are mean±SE from three independent experiments (N=3) with three plants in each experiment (n=3). *$p < 0.05$ by Student *t*-test.

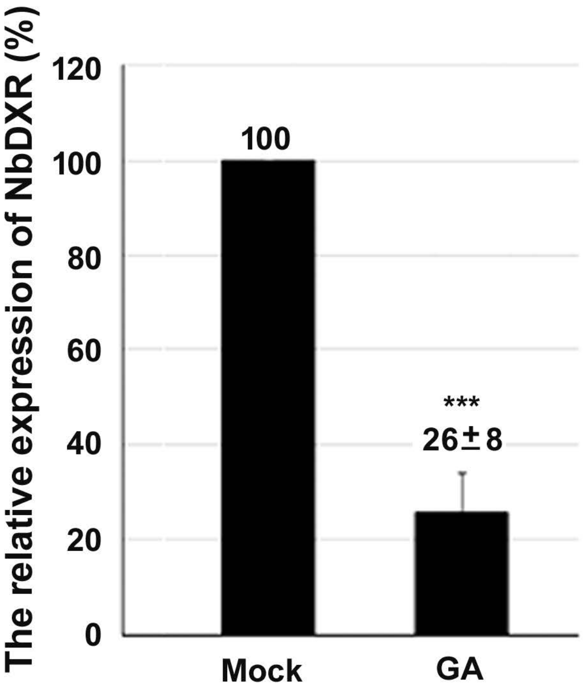

Fig. S8 The relative expression levels of *NbDXR* after GA treatment. The relative mRNA expression of *NbDXR* in mock and GA-treated protoplasts was determined by real-time quantitative RT-PCR at 24 hr post-inoculation. The expression of the *actin* gene was used for normalization. Data above bars are mean±SE from at least three independent experiments. ***$p < 0.001$ by Student *t*-test.
