# Supplementary material for: The gibberellic acid derived from the plastidial MEP pathway is involved in the accumulation of Bamboo mosaic virus
Source: New Phytol. 2022 Jun 22;235(4):1543–57. doi: 10.1111/nph.18210 (PMC9543464; doi:10.1111/nph.18210)
Supplement: Supplementary file 2 — Table S1 List of primer names, sequences, and purpose in this study. Please note: Wiley Blackwell are not responsible for the content or functionality of any Supporting Information supplied by the authors. Any queries (other than missing material) should be directed to the New Phytologist Central Office. [file NPH-235-1543-s002.pdf]

New Phytologist **Supporting Information**

Article title: **The gibberellic acid derived from the plastidial MEP pathway is involved in the accumulation of *Bamboo mosaic virus***

Authors: Ying-Ping Huang, I-Hsuan Chen, Yu-Shun Kao, Yau-Heiu Hsu and Ching-Hsiu Tsa

Table S1. List of primer names, sequences, and purpose in this study.

Primer name	Primer sequence	Primer usage	qPCR efficiency
NbDXR-F	5′-GATTCTTCCTGCAGATTCAGAACATT-3′	NbDXR expression detection	94%
NbDXR-R	5′-CCCCATGTTCCAATTGGGATGCTTCAAA-3′	NbDXR expression detection	
NbHDR-F	5'-CTCAAGCAACACTTCACATCTTCA-3'	NbHDR expression detection	90%
NbHDR-R	5'-GGTAAGAAGTTCTCTTTCTCGACC-3'	NbHDR expression detection	
NbGGPPS11-F	5′-ATCGTGAAAATTAGGA-3′	NbGGPPS11 expression detection	100%
NbGGPPS11-R	5′-CTTCTTTCTAAATCTTTTTCC-3′	NbGGPPS11 expression detection	
NbGGPPS2-F	5′-GAGAATGAGGAACTAT-3′	NbGGPPS2 expression detection	114%
NbGGPPS2-R	5′-GGCATCAATGTAGCTTAGCT-3′	NbGGPPS2 expression detection	
NbKS-F	5′-GCAACACCCCGAATA-3′	NbKS expression detection	104%
NbKS-R	5′-ACTACGCTGCCCTCT-3′	NbKS expression detection	
NbCA-F	5′-AGTGCATGTGGAGGTATCAAAGGT-3′	NbCA expression detection	91%
NbCA-R	5′-GTCGACTACGGAAAGAGAAGG-3′	NbCA expression detection	
NbFNR-F	5′-GCAGTTTCTCTTCCATCATCCAAGTCC-3′	NbFNR expression detection	101%
NbFNR-R	5′-CTCTGTGGTCACCTGGGCTCTG-3′	NbFNR expression detection	
actin-F	5′-GATGAAGATACTCACAGAAAGA-3′	actin expression detection	94%
actin-R	5′- GTGGTTTCATGAATGCCAGCA-3′	actin expression detection	
NbDXR-siF	5′-TAAGTGCAGCAAACGA-3′	NbDXR silencing	-
NbDXR-siR	5′-TCCCGAGCCCACAAAT-3′	NbDXR silencing	-
NbCMK-siF	5′-GGTTGTCTTGCCACTGAA-3′	NbCMK silencing	-
NbCMK-siR	5′-TGGAGAACTCGGCTTGAT-3′	NbCMK silencing	-
NbHDR-siF	5′-GGTTGGAACTCAAGCAACA-3′	NbHDR silencing	-
NbHDR-siR	5′-TGATTTACATTACTACAGAA-3′	NbHDR silencing	-
NbGGPPS11-siF	5′-’CGCGAGGTGTATT-3′	NbGGPPS11 silencing	-
NbGGPPS11-siR	5′-TAAGCAATGTAAT-3′	NbGGPPS11 silencing	-
NbGGPPS2-siF	5′-TGAACATGTAGCTT-3′	NbGGPPS2 silencing	-
NbGGPPS2-siR	5′-TCCACATCAATCT-3′	NbGGPPS2 silencing	-
NbKS-siF	5′-ATTGGAGAATTGAGCGGTA-3′	NbKS silencing	-
NbKS-siR	5′-TCCAAGTTTGTAAAATTAA-3′	NbKS silencing	-
NbDXR-T7F	5′-GTCTAGAATGGCGCTGAATTTGCTGTCA-3′	NbDXR with T7-tag	-
NbDXR-T7R	5′-GAGCTCTTA<u>GTCGACACCCATTTGCTGTCCACCAGTCATGCTAGCCAT</u>TACAAGAGCTGGACTAAAACC-3′	NbDXR with T7-tag; the sequence of T7-tag is underlined	-
NbDXR-OFPF	5′- GTCTAGAATGGCGCTGAATTTGCTGTCA-3′	NbDXR with OFP fusion	-
NbDXR-OFPR	5′-GGATCCTACAAGAGCTGGACTAAAACC-3′	NbDXR with OFP fusion	-
